# Effect of Different Membranes on Vertical Bone Regeneration: A Systematic Review and Network Meta-Analysis

**DOI:** 10.1155/2022/7742687

**Published:** 2022-07-14

**Authors:** Mi Zhang, Zili Zhou, Jiahao Yun, Rui Liu, Jie Li, Yimeng Chen, HongXin Cai, Heng Bo Jiang, Eui-Seok Lee, Jianmin Han, Yunhan Sun

**Affiliations:** ^1^Department of Dental Materials, Peking University School and Hospital of Stomatology, National Center of Stomatology, National Clinical Research Center for Oral Diseases, National Engineering Laboratory for Digital and Material Technology of Stomatology, Beijing Key Laboratory of Digital Stomatology, Research Center of Engineering and Technology for Computerized Dentistry Ministry of Health, NMPA Key Laboratory for Dental Materials, Beijing 100081, China; ^2^The Conversationalist Club, School of Stomatology, Shandong First Medical University & Shandong Academy of Medical Sciences, Jinan, Shandong 250117, China; ^3^Department and Research Institute of Dental Biomaterials and Bioengineering, Yonsei University College of Dentistry, Seoul 03722, Republic of Korea; ^4^Department of Oral and Maxillofacial Surgery, Graduate School of Clinical Dentistry, Korea University, Seoul 08308, Republic of Korea

## Abstract

This study is aimed at performing a systematic review and a network meta-analysis of the effects of several membranes on vertical bone regeneration and clinical complications in guided bone regeneration (GBR) or guided tissue regeneration (GTR). We compared the effects of the following membranes: high-density polytetrafluoroethylene (d-PTFE), expanded polytetrafluoroethylene (e-PTFE), crosslinked collagen membrane (CCM), noncrosslinked collagen membrane (CM), titanium mesh (TM), titanium mesh plus noncrosslinked (TM + CM), titanium mesh plus crosslinked (TM + CCM), titanium-reinforced d-PTFE, titanium-reinforced e-PTFE, polylactic acid (PLA), polyethylene glycol (PEG), and polylactic acid 910 (PLA910). Using the PICOS principles to help determine inclusion criteria, articles are collected using PubMed, Web of Science, and other databases. Assess the risk of deviation and the quality of evidence using the Cochrane Evaluation Manual, and GRADE. 27 articles were finally included. 19 articles were included in a network meta-analysis with vertical bone increment as an outcome measure. The network meta-analysis includes network diagrams, paired-comparison forest diagrams, funnel diagrams, surface under the cumulative ranking curve (SUCRA) diagrams, and sensitivity analysis diagrams. SUCRA indicated that titanium-reinforced d-PTFE exhibited the highest vertical bone increment effect. Meanwhile, we analyzed the complications of 19 studies and found that soft tissue injury and membrane exposure were the most common complications.

## 1. Introduction

Insufficient three-dimensional bone mass due to jaw surgery, trauma, tooth extraction, and age-related changes is a common problem in the field of dental implants, and sufficient bone mass is an important prerequisite for favorable prognosis in implant dentistry [1]. The guided bone regeneration (GBR)/guided tissue regeneration (GTR) technique is commonly used for bone regeneration in the alveolar ridge area. The barrier membrane plays an important role in bone regeneration during GBR/GTR. The main principle is to separate the bone defect from the surrounding connective tissue with a barrier membrane, prevent fast-growing soft tissue from growing into the bone defect, provide enough growth space for osteoblasts in the defect, and perform periodontal (bone) tissue repair [2]. The characteristics of ideal membranes for GBR/GTR treatment include biocompatibility, cell-occlusion properties, tissue integration, clinical manageability, space maintenance ability, and adequate physical properties [3].

Barrier membranes are generally divided into absorbable and nonabsorbable membranes based on their degradability [4]. The first barrier membrane used in GBR/GTR was expanded polytetrafluoroethylene (e-PTFE) [1], which has a strong ability to maintain the submembrane space and facilitate the growth of osteoblasts [5]. However, e-PTFE can be accompanied by more serious complications such as membrane exposure and varying degrees of bacterial infection, so high-density polytetrafluoroethylene (d-PTFE) was developed. As a substitute membrane for e-PTFE, d-PTFE has a low probability of bacterial infection, which can better protect the underlying bone graft material and make it easier to remove it in the second surgery [6]. In addition, metal-based membranes such as a titanium mesh (TM) are used in GBR/GTR due to the following characteristics: high stiffness, low density, and high temperature and corrosion resistance [1]. Titanium is added to polytetrafluoroethylene (PTFE) as a stabilizer to form titanium-reinforced PTFE. An absorbable membrane-covered TM and titanium-reinforced PTFE have been successfully used for vertical and horizontal bone regeneration around implants and are becoming commercially available. Despite the many advantages of nonabsorbable membranes, secondary surgical removal is inevitable and the increased risk of membrane exposure and bacterial infection remains [6].

Second-generation membranes made of absorbable materials have been developed to overcome the disadvantages of nonabsorbable membranes. For GBR/GTR, absorbable membranes are divided into two categories: natural and artificial polymer membranes. The most common natural polymer membranes are collagen membranes (CM), which have advantages that include low cost, no need for a second surgery for removal, and a lower risk of complications, such as membrane exposure and tissue damage [7]. Among them, Bio-Gide is the commonly used commercial collagen membrane. The dense layer of the membrane is close to the surrounding tissue surface, which has a good cellular barrier effect; the lax layer is close to the site of the bone defect area, which has more pores and plays a role in stabilizing the blood clot and facilitating the adhesion of the newly generated bone tissue to the membrane [8]. However, traditional collagen membranes are often unstable and may collapse into defects or deform under loading, resulting in impaired bone tissue regeneration [9]. Therefore, they are often used in combination with bone grafts to prolong degradation time and improve mechanical properties [10]. Furthermore, crosslinked collagen membranes (CCM) have been developed by modifying the absorbable membrane by changing its fibril orientation, thickness, or pore size. Despite prolonging the lifespan of the barrier membrane, certain chemicals (residual reagents or secondary products) may lead to an inflammatory response in the tissue, particularly during the degradation of the crosslinked membrane [11]. In addition, membranes made of artificial polymers, such as polylactic acid, polyglycolic acid (PLA), polyethylene glycol, and its copolymers, are used for GBR and GTR. However, they share similar disadvantages with collagen membranes, such as low stability and lack of rigidity. In addition, synthetic polymer membranes may induce significant inflammatory responses during degradation, which, in turn, may negatively affect bone or tissue regeneration [12].

Therefore, we performed a systematic review and network meta-analysis of studies related to maxillary bone grafting and analyzed the effectiveness of barrier membrane materials in GBR/GTR. We compared the osteogenic effects and complications of 11 barrier membranes: d-PTFE, e-PTFE, CCM, CM, TM, titanium mesh plus noncrosslinked (TM + CM), TM plus crosslinked membrane (TM + CCM), titanium-reinforced d-PTFE, titanium-reinforced e-PTFE, PLA, and polylactic acid 910 (PLA910). In addition to the network meta-analysis of the effect of different membrane materials on vertical bone regeneration, we also performed an analysis of the complications caused by different membrane materials, which is more clinically significant and differs from traditional network meta-analyses.

## 2. Materials and Methods

### 2.1. Study Selection

#### 2.1.1. Patients

Healthy patients using barrier membranes for vertical bone augmentation in GBR or GTR require dental implants to restore their oral function. There were no sex limits and smokers were excluded.

#### 2.1.2. Intervention

Different types of membranes and bone-filling materials are used in GBR and GTR.

#### 2.1.3. Comparator

Membranes are used in GBR or GTR. Different types of barrier membranes exist, including absorbable and nonabsorbable membranes.

#### 2.1.4. Outcomes

Vertical bone growth and the type and incidence of complications, including membrane exposure, infection or abscess, and soft tissue injury, among other outcomes, during the follow-up period after surgery, are the outcomes.

The specific inclusion and exclusion criteria are shown in Table 1.

### 2.2. Literature Screening

**Figure 1:**
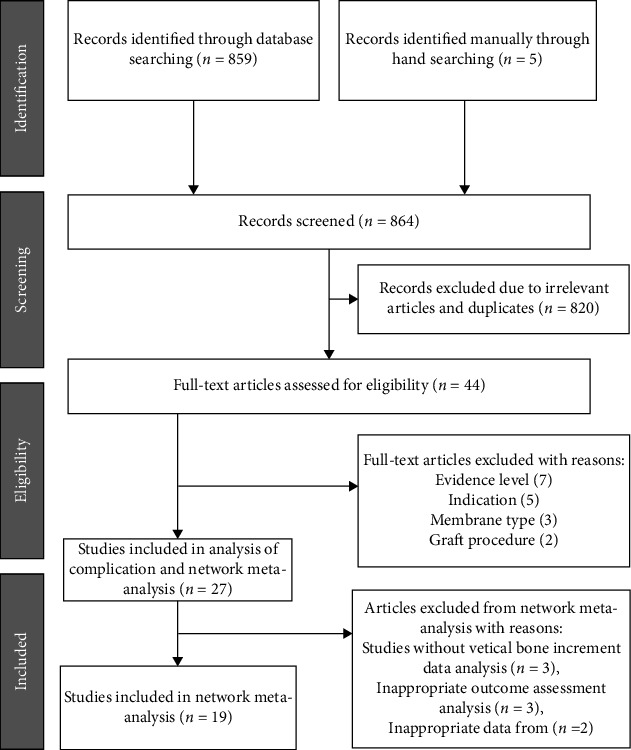
Preferred reporting items for systematic reviews and meta-analysis (PRISMA) flowchart of the search strategy for the systematic review.

Two researchers independently searched PubMed, Web of Science, Embase, Science Direct, Cochrane, and other databases for electronic literature, limited to articles published in English. For example, in PubMed, the searching mode was (“GBR”[All Fields] OR (“guided tissue regeneration”[MeSH Terms] OR (“guided”[All Fields] AND “tissue”[All Fields] AND “regeneration”[All Fields]) OR “guided tissue regeneration”[All Fields])) AND (“membranal”[All Fields] OR “membrane s”[All Fields] OR “membraneous”[All Fields] OR “membranes”[MeSH Terms] OR “membranes”[All Fields] OR “membrane”[All Fields] OR “membranous”[All Fields]) AND (“dentistry”[MeSH Terms] OR “dentistry”[All Fields] OR “dentistry s”[All Fields]) AND “randomized controlled trial”[Publication Type] AND “humans”[MeSH Terms]. The details are shown in Table 2. Subsequently, two independent reviewers evaluated the abstracts of the screened articles to exclude ineligible articles, and in case of disagreement, the opinion of a third reviewer was sought. After evaluating the abstracts, two reviewers performed a full-text analysis of eligible articles for final inclusion. At the end of full-text screening, the two reviewers exchanged notes and compared their selections to unify the screening criteria.  Figure 1 illustrates this specific process.

### 2.3. Research Data Selection and Extraction

Two authors independently extracted the general characteristics of the included studies, which were then recorded on a predesigned list. Extracted data included the following: types of membranes, number of patients in whom and sites where membranes were used, mean age and age range of patients, bone graft materials, follow-up time, vertical bone augmentation data, complications, and the first author of the original documents. The third author compared the data to ensure consistency.

### 2.4. Quality Assessment and Offset Risk

The data were extracted by the first author and included the following: year of publication, literature source, the country where the research institution was located, basic characteristics of the subjects included in the study, the intervention measures, the number of cases in the experimental and control groups, and the main and secondary outcome indicators. Based on the risk assessment tools recommended by the Cochrane Evaluation Manual (5.0.1), six aspects were evaluated for each included study, namely, selection bias, implementation bias, measurement bias, follow-up bias, report bias, and other biases. Each evaluation index was evaluated for three degrees of bias: low risk, unclear risk, and high risk, and reporting bias was evaluated using a funnel diagram in Stata SE (version 15.1; StataCorp, College Station, TX, USA).

Articles were classified by quality based on the Cochrane evaluation method. The selected literature was examined using the following seven indicators: generation of random sequences, allocation concealment, blinding of researchers and subjects, blind evaluation of research outcomes, completeness of outcome data, selective reporting of research results, and other sources of bias. If all the criteria were low risk, the study was judged as having a low risk of bias; if one or more criteria were unclear and there was no indicator of high risk, the study was judged as having an unclear risk of bias; and if one or more indicators were judged as high risk, the study was judged as having a high risk of bias. At the same time, the GRADE system was used to evaluate the included articles.

### 2.5. Data Analysis

Nineteen studies that met the inclusion criteria were included in the network meta-analysis (NMA), and vertical bone augmentation was selected as the outcome measure of the analysis. Additionally, the number of surgical sites included in the study were used as the unit of analysis. Combined effect sizes were calculated using the standardized mean difference (SMD), since vertical bone increments were measured differently in the included studies. At the same time, random effect models were used to explain the methodological differences between studies during the NMA. We used Stata SE (version 15.1; StataCorp, College Station, TX, USA) to perform global and local inconsistency analyses and to determine whether direct and indirect comparisons between subjects with different graft materials could be integrated. Heterogeneity was assessed by the chi-square test and *I*^2^ test, as well as pair comparison forest maps, with *I*^2^ ranging from 0% to 100% (the lower the value, the lesser the heterogeneity). A *P* value less than 0.05 indicated that heterogeneity was substantially reduced. The NMA includes network diagrams, paired-comparison forest diagrams, funnel diagrams, surface under the cumulative ranking curve (SUCRA) diagrams, and sensitivity analysis diagrams.

To evaluate each result, we used the SUCRA and average ranking. Additionally, we assessed the risk of bias in these studies based on the Cochrane Handbook for Systematic Reviews of Interventions.

The abovementioned NMA reports adhered to the PRISMA statement describing the data processing method.

## 3. Results

### 3.1. Analysis of the Included Studies

The detailed characteristics of the studies included are described in Table 3. In total, 27 overlapping papers were included. Among the 19 meta-analyses included, 18 were randomized controlled trials and 1 was a prospective trial. Four studies used a surgical approach to guide tissue regeneration and 15 studies used a surgical approach to guide bone regeneration. In total, 530 men and women aged 18–82 years were included in this study.

Eleven barrier membranes were included in this study: d-PTFE, e-PTFE, CCM, CM, TM, TM + CM, TM + CCM, titanium-reinforced d-PTFE, titanium-reinforced e-PTFE, PLA, and PLA910. Nonresorbable membranes were the most commonly used in the included studies (20 studies), including d-PTFE (two studies), e-PTFE (eight studies), TM (two studies), TM + CCM (two studies), TM + CM (one study), titanium-reinforced d-PTFE (two studies), and titanium-reinforced e-PTFE (three studies). The remaining membranes were absorbable membranes, divided into synthetic polymer-based absorbable membranes (four studies), and natural polymer-based absorbable membranes (15 studies). Synthetic polymer-based absorbable membranes included PLA 910 (two studies) and PLA (two studies). Resorbable membranes based on natural polymers included crosslinked (four studies) and noncrosslinked (11 studies) collagen membranes.

In the 19 included studies, autologous, allogeneic, and xenogeneic bone was used as a bone-filling material to restore the bone defect height. Autologous and allogeneic bones were used as bone-filling materials in the two studies. Autologous bone and xenograft bone, each accounting for 50%, were used as bone-filling materials in two studies. A mixed bone-filling material in which the allogeneic-bone-to-allogeneic-bone ratio was not specified was used in one study. Another study used a mixed bone-filling material without specifying the ratio of autogenous and xenogeneic bone. One, two, and three studies used autogenous, allogeneic, and xenogeneic bones alone, respectively. It is worth noting that another study used bovine bone as a bone-filling material in the control group and pig bone as a bone-filling material in the experimental group. Six studies did not specify the bone-filling materials used.

### 3.2. NMA

**Figure 2:**
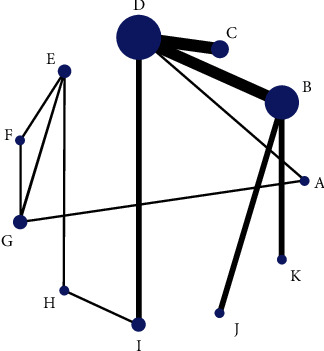
A network geometry plot. The plot presents the result of a network meta-analysis of the direct comparison of the 11 interventions. The width of the lines is proportional to the number of direct lines comparing every pair of interventions, and the size of every blue dot is proportional to the sample size of the interventions.

**Figure 3:**
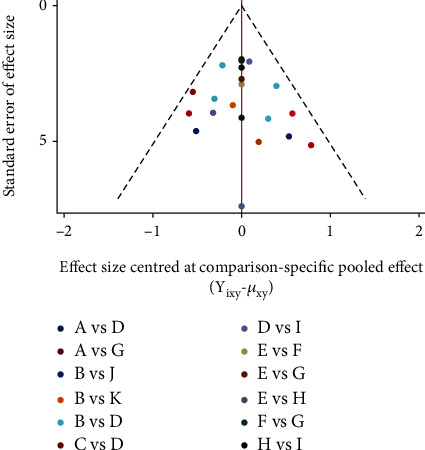
Funnel plot of publication bias. The horizontal axis represents the difference between the study-specific effect sizes from the corresponding comparison-specific summary effect. The vertical axis represents the standard error of the effect size. The red line represents the null hypothesis, in which the study-specific effect sizes do not differ from the respective comparison-specific pooled effect estimates.

**Figure 4:**
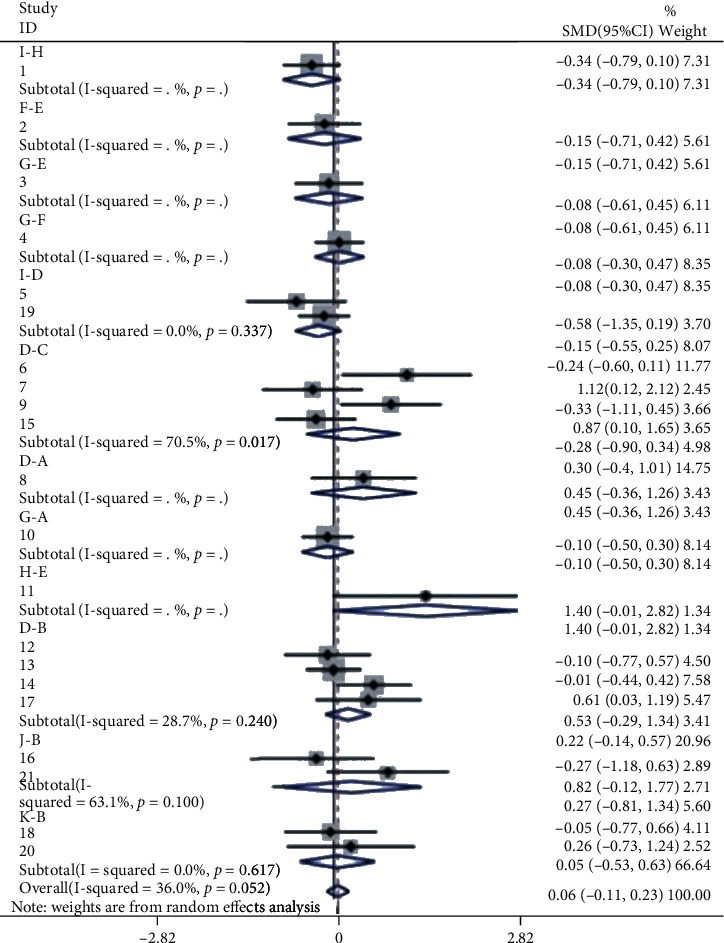
A forest map of pairwise comparison. A forest plot of pairwise comparison. The black horizontal lines represent the confidence interval (CI) of each study. The black solid diamonds represent the standard mean difference (SMD) for each study. The blue hollow diamond represents the result of pairwise comparison or the result of the entire study. The gray squares represent the weight of individual studies; therefore, the larger the sample size, the larger the weight and the larger the square area. The black vertical line in the middle is an invalid line.

**Figure 5:**
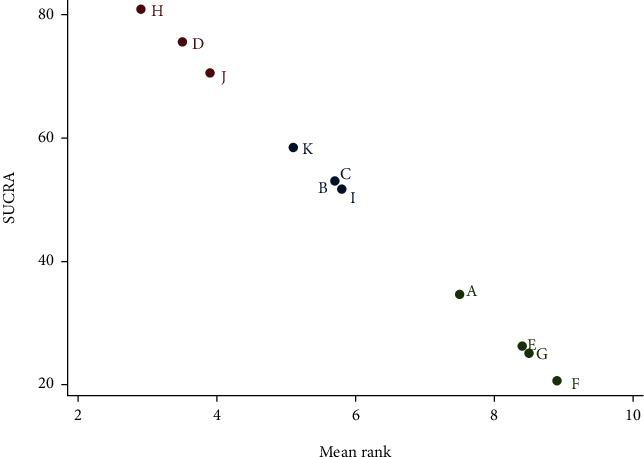
Plots of the SUCRA for all treatments included in this study. The SUCRA for all interventions that were included. The plot shows the percentage and ranking of the effectiveness of each treatment.

Firstly, Figure 2 shows the relationship between 11 different interventions, with each blue dot representing one intervention and each black line representing a direct comparison between the two interventions. Secondly, the plot shows that the area of the blue dot corresponding to the D group (CM) was the largest; therefore, the D group (CM) was the most frequent comparator among the studies. According to the fact that the width of the black line connecting the two blue dots is proportional to the number of the studies included that two directly compared interventions, the black line between B group (e-PTFE) and D group (CM) is the thickest, which represents the largest number of studies that directly compared interventions between B group (e-PTFE) and D group (CM). Simultaneously, this also happened in the directly compared interventions between the C group (CCM) and the D group (CM). In Figure 3, according to the meaning of points, each point represents a study, so bright-blue and red points appear the most frequently, which corresponds to the reason for the thickest black line between the B group (e-PTFE) and the D group (CM), or between the C group (CCM) and D group (CM) in Figure 2. Additionally, the dots of the funnel plot are based on the assumption that the accuracy of the effect size estimate increases as the sample size increases. Therefore, studies with small sample sizes are arranged symmetrically at the bottom of the plot and studies with large sample sizes are distributed at the top of the funnel plot and concentrated toward the middle of the plot. Most importantly, the dots reveal the absence of significant asymmetry and are all within the 95% confidence interval (CI). Consequently, there was little possibility of publication bias. Nevertheless, in Figure 4, when each 95% CI horizontal line in the plot intersects with the invalid vertical line (the abscissa scale is 0), it means that the corresponding interventions (membrane materials) in the experimental and control groups had similar application effects, indicating that there was no significant difference between the studies. In the forest plot, the overall *I*^2^ was 36.0% (<50%) and the *P* value was 0.052 (>0.05), indicating that there was no heterogeneity in the combined analysis among groups. The *I*^2^ in the compared interventions of the C group (CCM) and the D group (CM) was 70.5% (>50%) and the *P* value was 0.017 (<0.05), which provides evidence of statistical heterogeneity within the group. In addition, in the funnel plot, the red dots are distributed in very close proximity to the oblique dotted lines on either side, which also proves that the effect size of the comparison of the C group (CCM) and D group (CM) was statistically significant. Finally, in Figure 5, the H: titanium-reinforced d-PTFE (80.9%) has the best effect and F: TM + CM (20.6%) has the worst effect. Since the e-PTFE membrane has good biocompatibility and can protect against blood clots, it is regarded as the gold standard barrier functional material in clinical practice [13, 14]. Additionally, the blue dots corresponding to the B group (e-PTFE) and C group (CCM) appear to overlap. Nevertheless, the analysis results are slightly inconsistent, which may be related to the quality and quantity of the included literature. Hence, there were some limitations in the experimental results obtained in this study.

### 3.3. Inconsistency Test

We used Stata to conduct an inconsistency test of the statistical results. Based on Table 4, the results of the node-splitting method show that there was no statistical difference between the direct and indirect comparison results of any two membranes; therefore, we concluded that the results of our network meta-analysis were inconsistent and reliable.

### 3.4. Quality Evaluation

**Figure 6:**
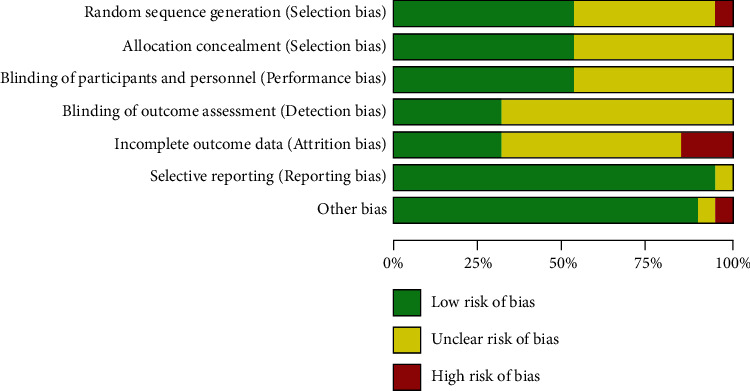
Risk of bias graph.

**Figure 7:**
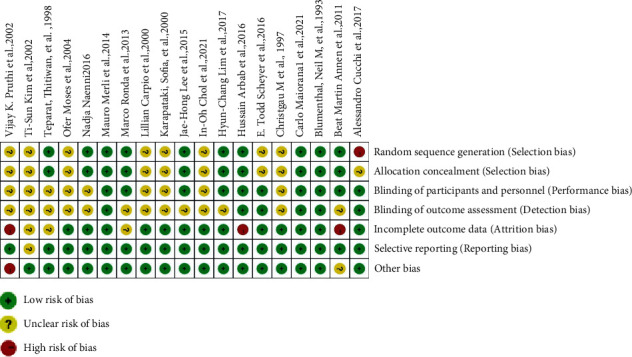
Risk of bias summary.

Figures 6 and 7 summarize the results of the quality assessment. All 19 studies were randomized controlled clinical trials (RCTs). A complete checklist (the Cochrane Collaborative Network tool for assessing bias risk) was used in the RCTs. Three studies had a low bias risk, and 12 studies had an unclear bias risk. Four studies had a high risk of bias. The quality assessments of various studies showed significant differences. Five studies showed that blindness was used, in which two were single blind and three were double blind.

### 3.5. GRADE Rating

Table 5 shows our confidence in the quality of evidence for pairwise direct comparisons and the ranking of treatments based on the GRADE approach. Our confidence ratings for pairwise direct comparisons were low and very low, mainly due to study limitations, inconsistency, and imprecision. In the overall treatment ranking, the confidence level in the quality of the evidence was low due to the limitations and imprecision of the included studies.

### 3.6. Sensitivity Analysis

**Figure 8:**
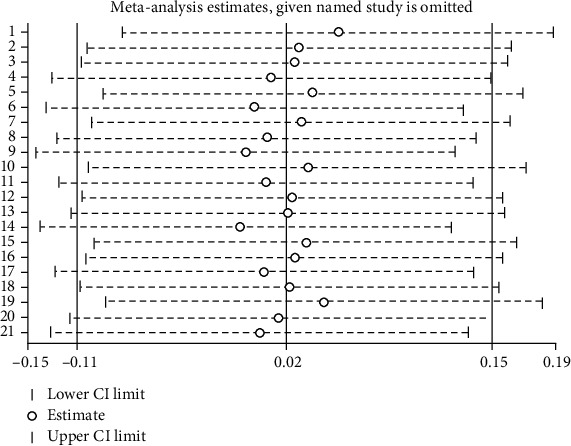
Influence of individual studies on overall results.

Figure 8 shows that the circles corresponding to the included 21 pairwise comparisons are all located near the middle vertical line where the combined effect size is located; therefore, it appears that there were no studies that had a significant impact on the combined effect size. The included articles were individually deleted, and no studies were identified that had a large effect on total heterogeneity.

## 4. Complication Analysis

**Figure 9:**
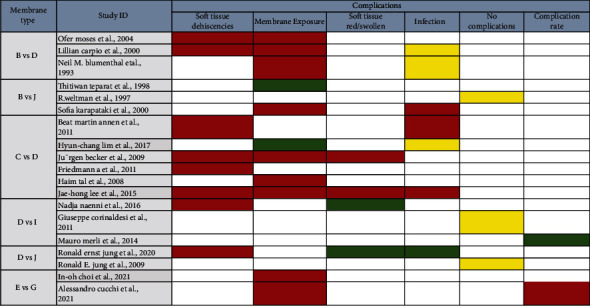
Results of the complication analysis. (red: number of control group occurrences > number of experimental group occurrences; green: number of experimental group occurrences > number of control group occurrences; yellow: there is no significant difference between the two groups).

The complications are shown in Figure 9.

### 4.1. B Group (e-PTFE) against the D Group (CM)

Three studies compared the e-PTFE group with the CM group [15–17].

Membrane exposure was reported in three studies, and the results showed that the e-PTFE group had a higher probability of membrane exposure [15–17]. Although the probability of wound dehiscence and membrane exposure was higher at the e-PTFE membrane site, the larger area of exposure at the collagen barrier site resulted in substantial implant exposure [16]. During the follow-up, membrane exposure occurred at nine sites (12 sites in total) in the e-PTFE group; however, no membrane exposure was found in the CM group [17]. When the collagen barrier is exposed prematurely, it is degraded by bacterial collagenase, thus jeopardizing the GBR results [15]. The e-PTFE has a layered structure and quickly becomes contaminated when exposed to the oral cavity, resulting in bacterial accumulation and infection [18].

Three studies described soft tissue healing, among which two studies showed a lower probability of soft tissue dehiscence in the CM group [15, 16]. Soft tissue inflammation was not significantly different between the two treatment modalities [16]. One study reported the absence of infection after either treatment [17].

### 4.2. B Group (e-PTFE) against the J Group (PLA)

Three studies compared the e-PTFE group with the PLA group [19–21].

Two studies described membrane exposure [19, 20]. One study showed that the PLA group performed better than the e-PTFE group. Teparat et al. reported that the coronal part of the e-PTFE barrier was exposed at 1 week and the exposure increased over time, while the exposure rate of the PLA film was lower [19]. One study showed that the e-PTFE group is superior to the PLA group. Three PLA-treated sites (10 in total) and two e-PTFE-treated sites (9 in total) developed soft tissue defects and membrane exposure in the first few weeks of healing [20].

One study described soft tissue inflammation, indicating that soft tissue inflammation occurs more frequently at the e-PTFE barrier treatment site and may be responsible for the accumulation of microorganisms between the flap tissue and the barrier [20].

One study described overall complications and showed that during a 9-month follow-up period, patients who received either treatment had no complications or significant discomfort [21].

### 4.3. C Group (CCM) against the D Group (CM)

Six studies compared the CCM group with the CM group [11, 22–26].

Membrane exposure was described in four studies [22–25]. One study showed that the membrane exposure rate in the CCM group (42%) was lower than that in the CM group (57%) during the follow-up [23]. Three studies showed that the membrane exposure rate was lower in the CM group than in the CCM group during follow-up [22, 24, 25]. One study reported re-epithelial perforation 4 weeks after membrane exposure, rapid disintegration of the CM membrane after exposure, and perforation in the CCM group, showing gradual migration of soft tissue to cover the membrane [24].

Soft tissue dehiscence was described in four studies [11, 22, 25, 26]. The results showed that the probability of soft tissue dehiscence was increased in the CCM group [11, 22, 25, 26]. This may be because the graft material lacks membrane support, leading to an increased probability of soft tissue dehiscence [26].

Two studies described redness and swelling of soft tissue [22, 25], and the results showed that the CCM group had a higher probability of redness and swelling of the soft tissue. Lee et al. reported that a patient in the CCM group had localized gingival redness, swelling, and discharge of pus at the 4-week examination [22].

Three studies described the infection status [11, 22, 23]. Two of these studies showed that CM membranes are less frequently infected [11, 22]. The membrane was removed in three cases (33%) due to severe infection in the CCM group and no severe infection in the CM group [11]. Another study reported no signs of infection in either treatment group [23].

### 4.4. D Group (CM) against the I Group (Titanium-Reinforced e-PTFE)

Three studies compared the CM group with the titanium-reinforced e-PTFE group [27–29].

One study described soft tissue redness with a lower probability of redness with CM (15.4%) than with titanium-reinforced e-PTFE (35.7%) [27].

A study described soft tissue dehiscence in the CM group in four cases (30%), titanium-reinforced e-PTFE groups in two cases (14%), and a low rate of dehiscence in the titanium-reinforced e-PTFE group [27].

One study reported a lower overall complication rate in the CM group (36%) than in the titanium-reinforced e-PTFE group (45%) [28].

One study found no complications in either group [29].

### 4.5. D Group (CM) against the J Group (PEG)

There were two studies comparing the CM group with the PEG group [30, 31].

One study described soft tissue healing [30]. The rate of soft tissue swelling was higher in the PEG group (29.7%) than in the CM group (26.8%). The rate of soft tissue cracking in the PEG group (7%) was lower than that in the CM group (14%) [30].

One study showed that no complications were found in either group [31].

### 4.6. E Group (TM) against the G Group (TM + CCM)

Two studies compared the TM group with the TM + CCM group [14, 32].

Two studies described membrane exposure, and both showed that the membrane exposure rate of the TM + CCM group was lower than that of the TM group [14, 32].

One study described the total complication rate, and the results showed that the TM + CCM group (13%) had a lower probability of complication than the TM group (33%) [14].

## 5. Discussion

The results of the network meta-analysis indicated that *I*^2^ was 36.0% (<50%) and the *P* value was 0.052 (>0.05), proving that the heterogeneity of the data in the included literature was low after merging and processing. In terms of the comprehensive analysis results of vertical bone regeneration, the titanium-reinforced d-PTFE membrane had the best comprehensive effect on vertical bone regeneration, whereas TM and TM + CM had no statistical difference in the comprehensive results of vertical bone regeneration, and both had a poor effect on vertical bone regeneration. Ronda et al. used 50% autologous bone and 50% mineralized allogeneic bone to fill the defect in prospective randomized controlled experimental surgery and reported that vertical bone regeneration was related to composite graft materials [33]. In a study by Ferrantino et al., 50% autologous bone and 50% deproteinized bovine bone mineral particles were used as bone graft-filling materials to compare the vertical bone gain of TM and titanium-reinforced d-PTFE [34]. In both prospective studies involving titanium-enhanced d-PTFE, titanium-reinforced d-PTFE membranes had better vertical bone regeneration than control membranes. However, the two studies included 23 and 5 patients, with relatively small sample sizes, which may have limitations. In a study by Choi et al., the vertical bone gain obtained using pure TM was not as good as that of the other two membranes. These authors used allograft bone as a bone graft material to fill the defect, and 100 people were included for statistical analysis; the results were more convincing [32].

Based on the SUCRA ranking probability plot, we can conclude that titanium-reinforced d-PTFE is the top-ranked barrier membrane, whereas TM + CCM is the bottom-ranked membrane. In a prospective randomized controlled trial, Ronda et al. used 50% autologous bone and 50% mineralized allogeneic bone to fill the defect and reported a relationship between the regeneration of vertical bone and the composite graft material. Another study examining titanium-reinforced d-PTFE was conducted by Maiorana et al. This study compared the vertical bone gain between TM and titanium-reinforced d-PTFE using 50% autologous bone and 50% deproteinized bovine bone mineral particles as bone graft-filling materials. Both studies that included titanium-reinforced d-PTFE concluded that the titanium-reinforced d-PTFE membrane was more effective than the control membrane in vertical bone regeneration. Since the porosity of the d-PTFE membrane is less than 0.3 microns, it may be impervious to bacterial penetration. In addition, the titanium framework allows the titanium-reinforced d-PTFE barrier membrane to be trimmed to a specific shape and form tents and space-maintained shapes [35]. However, a second surgery was required for removal. Nevertheless, the numbers of patients included in these two studies were 23 and 5, which is a relatively small sample size and may constitute a limitation of these studies. In our review, we identified a study with the greatest amount of vertical bone regeneration, specifically a vertical bone gain of 6.24 ± 2.98 mm. This randomized controlled trial used a crosslinked collagen membrane as a barrier membrane and a demineralized allograft as the bone-filling material. The maximum vertical bone augmentation obtained in this study may be due to the good resorbability of the CCM, which does not require secondary surgical removal, and its enhanced mechanical and biodegradable stability due to modification [35]. In the complication analysis, we can see that there were fewer complications in the titanium-reinforced d-PTFE membranes. The e-PTFE barrier membrane, which is considered the gold standard in other papers, did not perform well in this study, which may be related to the limited number of studies included [33].

GBR and GTR are commonly used techniques in the field of dental implants and periodontium [36]. Among these techniques, the barrier membrane plays an important role in good bone regeneration by separating soft tissue and bone defects. Due to the merits and shortcomings of various membranes, the gold standard for barrier membranes remains uncertain. Some studies suggest that the collagen membrane is the best biomaterial due to its good biocompatibility and biodegradability [37]. However, collagen membranes are prone to deformation due to their low strength, which affects bone regeneration. e-PTFE is the gold standard material with good biocompatibility and has significant bone regeneration effects in many clinical studies. The e-PTFE membranes may cause soft tissue splitting, leading to infection [38]. For multidimensional alveolar bone defects, some studies believe that titanium-reinforced PTFE, an absorbable material with high mechanical strength, is the gold standard in GBR/GTR surgery [3, 39]. However, nonabsorbable materials require a second surgery to be removed, which increases the patient's pain, treatment costs, and the probability of complications. Although existing reviews have summarized existing barrier membrane materials [36], there is a lack of comprehensive evaluation of their osteogenic effects and complications. This is the first comprehensive evaluation of several existing barrier membranes. The vertical bone increment of 11 barrier membrane materials was compared by a reticular meta-analysis, and the related complications were analyzed.

We included 19 papers, and in terms of literature screening, some gray literature may not have been retrieved even though we searched multiple mainstream literature repositories using a comprehensive search strategy. We carried out a sensitivity analysis, excluding some of the literature that has a greater effect on the overall effect and heterogeneity; therefore, for some membranes, the number of papers may be small. In addition, many studies have used vertical bone growth as a criterion for judging bone regeneration. Therefore, we used vertical bone increment as an outcome measure in this study.

The main function of the barrier membrane is to inhibit the migration of rapidly growing connective tissue to the defect and promote the growth of bone-side hard tissue. Current clinical research also focuses on improving osteoinduction and biocompatibility. Omar et al. found that cell and molecular activities in membranes are closely related to the promotion of bone regeneration and the addition of growth factors and cells to membranes or the use of graft materials may enhance the regeneration process of potential defects [40]. Zhang et al. developed a novel multifunctional GBR membrane with a specially designed porous layer to promote osteoblast adhesion [41]. Aprile et al. showed that the GBR membrane materials mainly used in the market and clinic are collagen membrane, TM, polylactic acid (PLA), e-PTFE, and d-PTFE [42]. However, there are few systematic reviews and meta-analyses on vertical bone gain and complications in GBR/GTR. The use of a barrier membrane is an effective method for protecting bone defects. We believe that in future studies, we can focus on the following aspects. First, appropriate selection of membrane materials and production methods may help induce new bone formation and the combination of multiple processes of biocompatibility of composite membranes has good development prospects. Moreover, clinical trials often differ in important areas, such as the location of the defect, protocol for administering the treatment, duration of treatment, and criteria for evaluation. The outcome of the surgical trial depends not only on the type of membrane but also on the surgeon's performance and expertise, as well as the patient's medical history and lifestyle, including oral hygiene and smoking. Therefore, the results of clinical trials should be compared indirectly through a rigorous meta-analysis. Finally, GBR/GTR technology is being developed to increase efficiency, individuation, and environmental protection. In future clinical applications, different membrane materials and production programs should be selected based on the clinical needs of each patient.

## 6. Conclusion

The vertical bone increment of several barrier membranes used in GBR/GTR was analyzed by a reticular meta-analysis, and the complications of several barrier membranes were analyzed. Based on this series of comprehensive analyses, it was determined that the application of a titanium-reinforced e-PTFE membrane in GBR/GTR may be the best choice. Regarding complications, membrane exposure and soft tissue complications are relatively common in GBR/GTR [43, 44], which is consistent with the following conclusions obtained in this study. Although our study has some limitations, our findings provide useful information for implementing GBR/GTR.

## Figures and Tables

**Table 1:** Inclusion and exclusion criteria.

	Inclusion criteria	Exclusion criteria
Language	English	Else
Study design	Randomized controlled trials/case series	Nonrandomized controlled trials (no control group); studies reported only in the following forms: retrospective studies, review articles, and literature reviews; publications using replicated information
Intervention	Comparison of two or more membranes	Studies involving only one membrane: the membrane studied is not in the range of the selected 7 membranes
Type of operation	GBR/GTR	Else
Surgical site	In the human body; in the mouth	Else
Method	Include patient selection criteria and relevant information; include a description of the procedure	The patient was younger than 18 The patient's physical condition does not match the criteria; the measurement method of vertical bone increment is not clear or does not match the unified standard (*n*)
Outcome	Includes results related to vertical bone gain; includes results related to complications	Excludes results related to vertical bone gain; complication-related results are not included

**Table 2:** Search strategies for PubMed.

Search subject	Strategy	Result
#1 Intervention	“membranal”[All Fields] OR “membrane s”[All Fields] OR “membraneous”[All Fields] OR “membranes”[MeSH Terms] OR “membranes”[All Fields] OR “membrane”[All Fields] OR “membranous”[All Fields]	1162158
#2 Position of study	“dentistry”[MeSH Terms] OR “dentistry”[All Fields] OR “dentistry s”[All Fields]	661109
#3 Type of study	“GBR”[All Fields] OR (“guided tissue regeneration” [MeSH Terms] OR (“guided”[All Fields] AND “tissue”[All Fields] AND “regeneration”[All Fields]) OR “guided tissue regeneration”[All Fields]	11476
#4 Article type	“randomized controlled trial”[Publication Type]	569132
#5 Object of study	“humans”[MeSH Terms]	20460648
#6 Combination of all search keywords	#1 AND #2 AND #3 AND #4 AND #5	369

**Table 3:** Characteristics of the included studies.

Study ID	Membrane	Patients (*n*)/implants (*n*)	No. of patients (mean age; range of age) (in years)	Bone graft materials	Follow-up time (months)	Vertical bone gain (mm/%)	GBR/GTR
Ronda et al. [33]	Titanium-reinforced, d-PTFE	12/38	23 (49.60/30–78)	Autologous bone and mineralized bone allograft (1 : 1)	6	5.49 ± 1.58 mm	GBR
	Titanium-reinforced e-PTFE	11/40				4.91 ± 1.78 mm	
Pruthi et al. [13]	e-PTFE	17/17	17 (56.50/35–75)	NA	12	1.00 ± 2.03 mm	GTR
	CM	17/17				0.81 ± 1.80 mm	
Moses et al. [15]	CM	28/53	28 (50.50/NA)	Autogenous bone chips mixed with either bovine bone mineral	6–8	75.11 ± 18.99%	GBR
	e-PTFE	17/34	17 (55.40/NA)			75.26 ± 24.36%	
Annen et al. [11]	CCM	9/9	9 (50.20/NA)	Bovine bone mineral granules	6	1.80 ± 1.60 mm	GBR
	CM	9/9				4.70 ± 3.30 mm	
Carpio et al. [16]	CM	23/23	48 (NA/NA)	Anorganic bovine bone plus; autogenous bone chips (1 : 1)	6	2.65 ± 0.61 mm	GBR
	e-PTFE	25/25				2.26 ± 0.66 mm	
Lee et al. [22]	CM	14/14	28 (53.30/31–75)	Xenograft bone substitutes	4	5.00 ± 2.50 mm	GBR
	CCM	14/14				2.90 ± 2.30 mm	
Scheyer et al. [35]	CM	19/19	40 (NA/18–70)	Demineralized allograft	6	6.24 ± 2.98 mm	GBR
	CCM	21/21		Deproteinized bovine bone mineral		5.29 ± 3.73 mm	
Arbab et al. [45]	CM	12/12	12 (53.00/25–73)	Cancellous allograft and bovine-derived xenograft	4	1.20 ± 1.50 mm	GBR
	d-PTFE	12/12	12 (52.00/30–73)			0.50 ± 1.60 mm	
Naenni et al. [27]	CM	13/13	27 (51.85/NA)	Demineralized bovine bone mineral	6	3.41 ± 2.33 mm	GBR
	Titanium-reinforced; e-PTFE	14/14				2.14 ± 2.06 mm	
Cucchi et al. [46]	d-PTFE	19/54	36 (52.00/NA)	Autogenous bone and bone allograft (1 : 1)	12	4.20 ± 1.00 mm	GBR
	TM + CCM	17/44				4.10 ± 1.00 mm	
Lim et al. [23]	CCM	12/12	12 (53.83/NA)	Collagenated porcine bone	4	−1.50 ± 3.00 mm	GBR
	CM	14/14	14 (48.14/NA)	Collagenated bovine bone		0.70 ± 1.80 mm	
Maiorana et al. [34]	Titanium-reinforced; d-PTFE	5/5	5 (54.20/38–65)	Autogenous bone and deproteinized bovine bone mineral particles (1 : 1)	8	4.20 ± 2.20 mm	GBR
	TM	5/5				1.50 ± 1.60 mm	
Teparat et al. [19]	e-PTFE	10/9	10 (NA/40–65)	NA	9	3.30 ± 1.70 mm	GBR
	PLA	10/10				2.90 ± 1.20 mm	
Blumenthal [17]	e-PTFE	12/12	12 (NA/31–80)	NA	12	1.00 ± 1.04 mm	GBR
	CM	12/12				1.58 ± 1.16 mm	
Christgau et al. [47]	PLA 910	NA/16	11 (40.40/23–59)	NA	12	5.50 ± 1.50 mm	GTR
	e-PTFE	NA/14				5.60 ± 2.20 mm	
Merli et al. [28]	CM	11/42	11 (44.60/29–59)	Autologous bone	72	0.58 ± 0.66 mm	GBR
	Titanium-reinforced; e-PTFE	10/55	10 (49.90/36–69)			0.49 ± 0.53 mm	
Kim et al. [48]	PLA 910	8/8	8 (NA/NA)	NA	6	3.00 ± 1.70 mm	GTR
	e-PTFE	8/8				2.60 ± 1.40 mm	
Karapataki et al. [20]	PLA	10/10	19 (43.00/NA)	NA	12	4.40 ± 1.70 mm	GTR
	e-PTFE	9/9				3.00 ± 1.70 mm	
Choi et al. [32]	TM	15/17	100 (57.10/20–82)	An allograft bone	6	3.20 ± 1.70 mm	GBR
	TM + CCM	56/72				3.10 ± 1.20 mm	
	TM + CM	29/40				3.00 ± 1.20 mm	
Friedmann et al. [26]	CCM	17/37	37 (NA/24–69)	A coagulum was formed by the calcium phosphate grafting material	6	/	GBR
	CM	20/36					
Tal et al. [24]	CCM	52/52	52 (46.00/20–70)	Deproteinized bovine bone mineral	6		GBR
	CM	52/52					
Weltman et al. [21]	PLA	30/16	30 (NA/26–64)	NA	12		GBR
	e-PTFE	30/14					
Atef et al. [49]	CM	20/10	20 (NA/20–60)	Autogenous and anorganic bovine bone mineral bone mixture (1 : 1)	6		GBR
	TM	20/10					
Jung et al. [30]	PEG	57/57	117 (48.70/19–77)	A synthetic bone filler	6		GBR
	BG	57/57					
Jung et al. [31]	PEG	19/19	19 (48.00/32–72)	A natural bone mineral of bovine origin	6		GBR
	CM	18/18	18 (54.00/23–80)				
Cucchi et al. [14]	TM	15/34	30 (NA/NA)	Autogenous bone and bone xenograft (1 : 1)	6		GBR
	TM + CCM	15/37					
Becker et al. [25]	CCM	23/41	23 (44.90/NA)	Natural bone mineral	4		GBR
	CM	26/37	26 (42.40/NA)				

**Table 4:** Node-splitting analysis of inconsistency.

Side	Direct		Indirect		Difference		
	MD	SE	MD	SE	MD	SE	*P*
d-PTFE vs CM	0.4357833	0.5289967	1.163483	1.104742	−0.7277	1.224864	0.552
d-PTFE vs TM + CCM	−0.992163	0.3874848	−0.8464555	1.17107	0.7472392	1.233511	0.545
e-PTFE vs CM	0.2239182	0.2175388	0.7504707	14.15419	−0.5265525	14.15584	0.970
e-PTFE vs PLA	0.2455775	0.3966155	−0.7071713	44.73296	0.9527488	44.73485	0.983
e-PTFE vs PEG	0.0640996	0.369438	−0.6947047	44.73486	0.7588043	44.73644	0.986
CCM vs CM	0.2286887	0.2530728	1.142997	31.63677	−0.9143078	31.63774	0.977
CM vs titanium-reinforced e-PTFE	−0.3001702	0.3116784	0.4459553	1.193876	−0.7461255	1.233879	0.545
TM vs TM + CM	−0.155983	0.4390793	1.3408	2.415155	−1.496783	2.468483	0.544
TM vs TM + CCM	−0.0779806	0.4261512	0.6701218	1.158239	−0.7481025	1.234139	0.544
TM vs titanium-reinforced d-PTFE	1.244603	0.8095491	0.4960574	0.9320917	0.7485455	1.234571	0.544
TM + CM vs TM + CCM	0.0779914	0.3843957	1.57247	2.441477	−1.494478	2.467023	0.545
Titanium-reinforced e-PTFE vs titanium-reinforced d-PTFE	−0.3406294	0.40123	−1.088411	1.167402	0.7477821	1.234428	0.545

**Table 5:** GRADE summary of randomized controlled clinical trials included in the final analysis.

Comparison	Certainty	Downgrading due to
A vs D	⨁⨁◯◯, low	Study limitation, imprecision
A vs G	⨁⨁◯◯, low	Study limitation, imprecision
B vs D	⨁⨁◯◯, low	Study limitation, imprecision
B vs J	⨁⨁◯◯, low	Study limitation, imprecision
B vs K	⨁⨁◯◯, low	Study limitation, imprecision
C vs D	⨁◯◯◯, very low	Study limitation, inconsistency, imprecision
D vs I	⨁⨁◯◯, low	Study limitation, imprecision
E vs F	⨁⨁◯◯, low	Study limitation, imprecision
E vs G	⨁⨁◯◯, low	Study limitation, imprecision
E vs H	⨁⨁◯◯, low	Study limitation, imprecision
F vs G	⨁⨁◯◯, low	Study limitation, imprecision
H vs I	⨁⨁◯◯, low	Study limitation, imprecision
Ranking of treatments	⨁⨁◯◯, low	Study limitation, imprecision

## Data Availability

The data used to support the findings of this research are included within the article and are labeled with references.
